# Effects May Both Increase or Decrease in the Long Term: A Statistical Illustration

**DOI:** 10.1177/17456916261452937

**Published:** 2026-07-30

**Authors:** Mathias Berggren, Jessica Kay Flake

**Affiliations:** 1 Department of Psychology, Uppsala University; 2 Department of Psychology, University of British Columbia

**Keywords:** effect size, explained variance, small effects, practical significance

## Abstract

It has been argued that small effect sizes observed at a single instance should not be dismissed because they can often accumulate in the long term. This follows a classic argument by Abelson (1985). However, it has also been argued that such long-term accumulation is entirely speculative and need not happen. Using a simple statistical illustration, we show how restrictive Abelson’s assumptions are and argue that they are unlikely to be met in psychological research. This demonstrates that the Abelson example is unrealistically biased toward the accumulation of effect sizes; even small deviations can have large consequences for the long-term effect sizes. This is true even if no long-term counteracting mechanisms, such as habituation, are at play. Further, it is shown how long-term effect sizes may be smaller than those observed at a single time point. If the theoretical value of an effect lies in the long term, it should be examined in that long term, and not merely be the subject of speculation in the short term.

## Introduction

In 1985, Robert Abelson conducted a now-classic analysis by using major league baseball players’ batting averages to predict their results for a single bat, yielding what to him was a surprisingly low explained variance of .00317. The resolution that Abelson provided to this seeming paradox was that such apparently small effects of batting averages could accumulate in the long term (over multiple realizations of the effect, i.e., over multiple bats), meaning that even a small batting-average difference between players could ultimately be highly consequential.

Several authors have more recently taken up this reasoning to argue that small psychological effects can accumulate to be highly consequential in the long term (e.g., Funder & Ozer, 2019; Götz et al., 2022; Greenwald et al., 2015; see also examples in Anvari et al., 2023; Primbs et al., 2023).

This reasoning has drawn the criticism that such accumulation is entirely speculative, with no guarantee that it will happen for a specific effect (Anvari et al., 2023; Primbs et al., 2023; Sauer & Drummond, 2020). Although several theoretical criticisms exist, we have found no statistical derivation of this limitation. Here, we present a simple derivation, with accompanying examples and discussion. We focus on variance explained as in Abelson (1985), and we refer to small effects as those closer to zero than expected (or desired) by researchers, whether that expectation is based on some common standards, some external objective criteria, or other effect sizes in the literature. When deriving how the effects accumulate, it becomes apparent that accumulation like that in the Abelson (1985) example is unlikely to ever occur for any theoretically meaningful relationships between psychological variables. We also demonstrate that in straightforward situations the opposite of accumulation can occur: Effects are attenuated in the long term.

First, we review psychological literature on explained variance when measured across multiple time points and trials and discuss how the argument for long-term accumulation differs from typical long-term change models. Next, we review the Abelson (1985) example and its use in arguing for accumulation. We sketch different ways in which this example requires an unrealistically optimistic model of long-term accumulation. Specifically, we show statistical proof that the Abelson example relies on highly restrictive assumptions unlikely to be fulfilled for any psychological effects. In particular, it relies on the assumptions that the effect is causal, that there are no other stable causal effects predicting the dependent variable, and that the effect does not vary within participants.

Critically, in the Abelson example, the long-term predictor and outcome are one and the same (single bats add up to a batting average in the long term). As we will illustrate, even a small deviation from this assumption can dramatically alter the degree of accumulation when the original effect size is small. We finalize with a discussion on implications.

### Common models versus Abelson’s accumulation argument

Several commonly used statistical models are designed to assess slopes and explained variances in data measured over multiple time points, such as growth-curve models (Duncan et al., 2013), time-varying effect models (Tan et al., 2012), and, more broadly, multilevel models (Hoffman & Rovine, 2007; Rights & Serba, 2019, 2021). In repeated multilevel models, variance is typically partitioned into between-person variance and within-person variance, which together add up to a total variance (see Rights & Serba, 2019, 2021). Typical measures of explained variances focus on the total variance in the dependent variable (Lorah, 2018; McNeish & Hamaker, 2020; Nagakawa & Schielzeth, 2013; Wu et al., 2009). Researchers thus often want to quantify how precisely they can predict the total variation for the studied variables. One reason for focusing on total variation is that if researchers can show that a change in an independent variable always tends to be associated with a change in the dependent variable, this can provide stronger evidence for a causal relationship between variables.

Abelson observes a seeming paradox: The true batting average, the “skill” of players, explains only about .00317 of the *total* variance in the results of single bats (scored 0 for a miss and 1 for a hit; pp. 130–131) even though “‘everyone knows’ that the variable in question has substantial explanatory power” (p. 132). He resolves the paradox by considering how skill level has an influence over multiple bats and can therefore accumulate in the long term (p. 133). Abelson thus finalizes his treatment by noting that “one should not necessarily be scornful of miniscule values for percentage variance explained,” and that his baseball example is “a model for similar paradoxes that may arise in psychological contexts” (p. 133). This has become a recurring reference in articles making the argument for accumulation (Funder & Ozer, 2019; Götz et al., 2022). The resulting accumulation argument is that (in the absence of observing the long-term effect) the effect size can often be expected to be strong in the long term even if it seems weak in the short term.

The accumulation argument differs from the common approach of explaining total variance in a critical way. It relies on comparing long-term effects, that capture only stable between-person variation, to effects found in single trials, that are affected by the total variation across persons and trials. Funder and Ozer (2019) stated, “If a stable trait [. . .] affects much of what you do even in a small way, its consequences can add up quickly [. . .] A correlation of about .05 translates to large consequences with 550 at bats. How long does it take for a person to experience, for example, 550 interpersonal encounters?” (p. 161). The argument, then, is that any single realization of the outcome (successful bats, successful social encounters) might be poorly predicted by the independent variable (e.g., batting ability, a stable trait). That is, a small proportion of the total variation may be explained. But when the influence of that predictor is stable, then other unsystematic variations between the realizations will tend to cancel out and the predictor will come to explain a larger proportion of the remaining (between-person/long-term) variation.

But is the Abelson example a good model for accumulation of psychological effects? Next, we review some long-term dynamics that complicate such accumulation arguments. Then we will clarify our model and show that even if such long-term dynamics do not come into play, the Abelson example is still overly optimistic about the accumulation.

### Mechanisms that affect long-term accumulation

To fully understand long-term effects, one should consider the long-term mechanisms that can either amplify or counteract effects at a single time point (Anvari et al., 2023; Primbs et al., 2023). Any single time point of data can fail to account for meaningful long-term mechanisms that will moderate how the effect size develops. Anvari et al. (2023) list one amplifying mechanism—accumulation through repetition—and three counteracting mechanisms—habituation, counteracting responses, and homeostasis (p. 505).^1^ Abelson’s argument requires accumulation through repetition; if a variable’s effect repeats frequently, then there is more ground for an effect to accumulate meaningfully over a reasonable span of time. It does not include counteracting mechanisms like habituation. Habituation occurs when an effect becomes smaller over time because of a reduced response to that variable, such as when eyebrow-raising commercials may lose their effect over time.

Another way to frame the points above is to note that if researchers have studied an effect at a single time point then they can fail to account for aspects that interact with their favored variables; these aspects may either amplify or counteract the effects. If the independent variable’s effect at the dependent variable is studied for some certain time point but the actual effect is more delayed, then its true effect may be missed. And on the other hand, the immediate effect may be larger than the (perhaps more interesting) delayed one.

Two additional theoretical aspects remain important for accumulation: causality and measurement error. If there is no causal effect, then it cannot truly be accumulating in the long term. Beyond establishing correlation at a single time point, accumulation thus also requires establishing causality. Additionally, measurement error can lead to the effect being estimated as stronger or weaker than it is in truth. If measurement errors are entirely random and unbiased, then this will attenuate correlations between pairs of variables (Spearman, 1904). However, under broader and more realistic models of measurement error, this need not be the case. If the measurement introduces common method bias (Podsakoff et al., 2003), or careless responding (Stosic et al., 2024), then this may cause associations to appear stronger than they truly are (Clifton, 2020; Sauer & Drummond, 2020; and see Loken & Gelman, 2017).

### Should we assume substantial accumulation?

So far, we have presented conceptual arguments and limitations of long-term accumulation. Still, researchers who have measured a causal effect with little error, and which convincingly repeats over time, may still consider it the responsibility of critics to establish sufficient reason for nonaccumulation rather than putting the onus on themselves to establish accumulation. Abelson’s (1985) example seems to show that such effects can be expected to accumulate substantially in the long term.

The arguments by previous authors (Anvari et al., 2023; Primbs et al., 2023; Sauer & Drummond, 2020) show the importance of estimating an effect in its proper context with potential long-term dynamics. Next, we will show that the Abelson (1985) example is too optimistic about the accumulation of psychological effects even in the ideal case that it is a causal effect, measured properly, and that there are no complicating long-term mechanisms, such as habituation.

## Model of Long-Term to Short-Term Explained Variance

Here, we provide an overview of our model of long- and short-term effects to examine how the explained variance (as considered in Abelson, 1985) can vary in the long and short term. We skip the mathematical details here, but derive them in the Supplemental Material available online (see Appendix A). In the next section, we apply this model to the Abelson (1985) example. In subsequent sections, we illustrate how the additional terms in the model are generally required to capture accumulation across time points. An overarching example with coin flips is also derived in detail in Appendix B in the Supplemental Material.

We consider the optimal (least-squares) population model $M_{t}$ , equal to the true mean of the dependent variable $Y_{t}$ for each level of the independent variables at time point t , so that $Y_{t}=M_{t}+\varepsilon_{t}$ —where the error $\varepsilon_{t}$ is uncorrelated with the model. The population model is the same as the true model that researchers try to estimate. So we ask, if the true explained variance of the model was .00317, how would this accumulate in the long term, under various conditions?

Note that our notation works for multiple research designs. $M_{t}$ could, for example, be a simple linear model measured at a single time point, or some more complicated nonlinear function of the independent variables, or a longitudinal model. The independent variables could be measured at the same time point, some baseline time point, or multiple previous time points. Similarly, the dependent variable $Y_{t}$ could represent a baseline variable at time point t (e.g., GPA), or some transformation (e.g., difference in GPA from time point t−1 to t ). Our setup is thus very general, although we will mostly consider the setup that appears to be most common in studies arguing for substantial accumulation—that is, when a linear model is measured at a single time point.

When measuring accumulation, we consider the mean of the dependent variable over multiple time points.^2^ Let $Y_{t}$ denote the outcome for a single trial t . Let the long-term outcome ${\bar{Y}}_{1:T}$ denote the mean outcome over trials 1 to *T*, that is, ${\bar{Y}}_{1:T}=\frac{1}{T}\sum_{t=1}^{T}Y_{t}=\frac{1}{T}\sum_{t=1}^{T}\left(M_{t}+\varepsilon_{t}\right)=\frac{1}{T}\sum_{t=1}^{T}M_{t}+\frac{1}{T}\sum_{t=1}^{T}\varepsilon_{t}$ . We see that the long-term outcome ${\bar{Y}}_{1:T}$ can be expressed as a linear function of the mean of the model over those time points $\frac{1}{T}\sum_{t=1}^{T}M_{t},$ plus the mean of the error $\frac{1}{T}\sum_{t=1}^{T}\varepsilon_{t}$ .

The model and error terms can each be split into one stable part that remains even as the mean is taken over an increasing number of trials, plus one unstable part that cancels out more and more as the number of trials increases. This works similarly as for the reliability of measurements (e.g., of items in a scale), where measured scores consist of one stable part and one random part that cancel out as the number of measurements increases (Novick, 1966). In multilevel models with repeated measurements, this corresponds to the between-person variance (the stable part) and the within-person variance (the unstable part; Hoffman & Rovine, 2007; Rights & Sterba, 2019, 2021).

Call the stable parts that remain in the long term $M_{long}$ and $E_{long}$ for the model and error part, respectively, and call the unstable parts that cancel out in the long term $M_{t,short}$ and $E_{t,short}$ (see also Table 1). Long-term variables represent average tendencies of participants (or other units of study). Short-term variables represent the parts that vary within participants from one trial to the next. Only the short-term parts need t -subscripts because the long-term variables are stable across time points. We can now write the model for $Y_{t}$ as $Y_{t}=M_{long}+M_{t,short}+E_{long}+E_{t,short}$ . Let $\sigma_{Y_{t}}^{2}$ and $\sigma_{Y_{1:T}}^{2}$ denote the variance of $Y_{t}$ and ${\bar{Y}}_{1:T}$ across participants. It is then easy to derive (see Appendix A) that the variance across participants for a single time point must fulfill $\sigma_{Y_{t}}^{2}=\sigma_{M_{long}}^{2}+\sigma_{M_{t,short}}^{2}+\sigma_{E_{long}}^{2}+\sigma_{E_{t,short}}^{2}$ .

**Table 1.** Sources of Variation in the Outcome

	Stable part	Unstable part
Modeled part	$M_{long}$	$M_{short}$
Unmodeled part	$E_{long}$	$E_{short}$

Note: In repeated multilevel models, variables with long subscripts capture between-person variance, and variables with short subscripts capture within-person variance.

We can now derive a formula for the explained variance for the single trial. Because the variance of the modeled variables is explained, whereas the variance of the error is not, the true explained variance $R_{t}^{2}$ of the model in trial or time point t equals

(1) $$\begin{array}{c} R_{t}^{2}=\frac{\sigma_{M_{long}}^{2}+\sigma_{M_{t,short}}^{2}}{\sigma_{M_{long}}^{2}+\sigma_{M_{t,short}}^{2}+\sigma_{E_{long}}^{2}+\sigma_{E_{t,short}}^{2}} \end{array}$$

As the number of trials T increases (i.e., tends to infinity, denoted by ∞ ∞ ), the short-term parts cancel out and their variances go to zero. Thus, the explained variance of ${\bar{Y}}_{1:}$ ∞, denoted $R_{1:}^{2}$ ∞, tends toward

(2) $$\begin{array}{c} R_{1:\infty }^{2}=\frac{\sigma_{M_{long}}^{2}}{\sigma_{M_{long}}^{2}+\sigma_{E_{long}}^{2}} \end{array}$$

The explained variances in Equations 1 and 2 correspond to the total- and between-person variance explained in repeated multilevel models (Rights & Sterba, 2019; see also Shaw et al., 2023, for a tutorial). The total variance explained in Equation 1 is the variance between persons (long subscripts) plus the variance within person (short subscripts). For substantial accumulation of the explained variance to occur, Equation 2 should be substantially larger than Equation 1.

For simplicity, we will restrict our illustrations to cases in which the short-term effects are the same for each trial and the trials are independent of each other. This is not very consequential for our conclusions about the long-term limit (see Appendix A), but it is useful for illustrative purposes, because it means that the variances from short-term variables decrease by a factor 1/T as the number of trials T increases (see Appendix A).

We can now start applying this model. First, we use it to illustrate the lack of realism of the Abelson (1985) example, when applied, to understand the accumulation of psychological effects. Then we show how even small modifications can dramatically alter the accumulation of a small short-term effect size.

## Applications

### Application to the Abelson (1985) example

In the Abelson (1985) baseball example, the model of the independent variable $M_{t}$ equaled the true batting average of players—also denoted as their “skill level” by Abelson (e.g., p. 131)—whereas the (very) long-term outcome ${\bar{Y}}_{1:}$ ∞ referred to the mean of an infinite series of single bats, scored 0 for a miss and 1 for a hit (p. 130). In this specific case, the predictor and the long-term outcome are therefore the same, $M_{t}={\bar{Y}}_{1:\infty }$ .^3^ In practice, Abelson looked at “a period long enough to give a reliable indication of the batters’ true averages” (p. 130) and treated this as essentially the numbers that would be found for an infinite series of bats. For a single trial, the result of one bat $Y_{t}$ is similarly predicted by players’ true batting averages $M_{t}={\bar{Y}}_{1:\infty }$ .

The problem is this: When Abelson called average hit rate the “skill” of a player, this produced an apparent substantial difference between the predictor (independent variable) and the outcome (dependent variable), even though they were one and the same (at least over time).^4^ But when psychologists argue that their effects are likely to accumulate, it is the rule rather than the exception that the independent and dependent variables have some substantial differences. For example, psychological researchers are rarely interested in predicting the average rate of successful social interactions with a person’s true probability of having a successful social interaction. Rather, they might predict the average rate of successful social interactions with a separate construct, such as agreeableness (see Funder & Ozer, 2019).

Abelson’s modeled variable equals the long-term outcome, $M_{t}={\bar{Y}}_{1:\infty }$ , so there is no long-term variance other than the modeled one. That is, the unmodeled variance in the long term $\sigma_{E_{long}}^{2}$ equals 0. The long-term explained variance therefore reduces to

(3) $$\begin{array}{c} R_{1:\infty }^{2}=\frac{\sigma_{M_{long}}^{2}}{\sigma_{M_{long}}^{2}+\sigma_{E_{long}}^{2}}=\frac{\sigma_{M_{long}}^{2}}{\sigma_{M_{long}}^{2}}=1 \end{array}$$

or 100% explained variance. In other words, the amount of long-term explained variance cannot be any more favorable than it is in the Abelson example.

Predicting with the true batting average further means that no short-term variation in the modeled variable fails to generalize to the long term (the model already equals the long-term outcome), so $\sigma_{M_{short}}^{2}equals\;0$ . Consequently, the explained variance in the short term reduces to

(4) $$\begin{array}{c} R_{t}^{2}=\frac{\sigma_{M_{long}}^{2}+\sigma_{M_{short}}^{2}}{\sigma_{M_{long}}^{2}+\sigma_{M_{short}}^{2}+\sigma_{E_{long}}^{2}+\sigma_{E_{short}}^{2}}=\frac{\sigma_{M_{long}}^{2}}{\sigma_{M_{long}}^{2}+\sigma_{E_{short}}^{2}}\; \end{array}.$$

The low explained variance $\left(R_{t}^{2}=.00317\right)$ is due to the fact that the short-term unmodeled variance $(\sigma_{E_{short}}^{2}$ ; the within-person variance in one-time batting results) is much greater than the modeled long-term variance ( $\sigma_{M_{long}}^{2}$ ; the between-person variance in batting averages). Indeed, as the averages had a limited range from about .20 to .30, whereas the result of a single bat ranges from 0 to 1, this is necessarily so.

The Abelson example therefore works to produce substantial accumulation exactly because the long-term dependent variable has a single causal determinant equal to the independent variable (they are the same), whereas the single-trial dependent variable contains a lot of entirely random (short-term) variation that cancels out in the long term.

### Application to psychological variables

Is the Abelson (1985) formulation reasonable for understanding the long-term relationship between psychological variables? As noted above, most psychological phenomena are more causally complex and multiply determined than the relationship between average hit rates over a set number of bats and over all bats, respectively. Take the simplified example from Funder and Ozer (2019) where money spent on holiday shopping is predicted by extraversion. Here, the long-term outcome (money spent over multiple holidays) will depend on something other than the modeled variable (extraversion), provided there are other variables responsible for variation in holiday spending that are also relatively stable across time points (e.g., income).

Götz et al. (2022) argued in favor of accumulation but also acknowledge the complexity of psychological variables, including the fact that personality is affected by factors such as genetics, childhood experiences, family environments, major life events, neighborhood characteristics, climate, evolutionary processes, and culture (p. 206). Indeed, it has long been argued that “variables employed in psychological research [. . .] [tend to be] affected by the interaction of a vast number of factors” (Lykken, 1968, p. 153). But the Abelson example does not take into account this complexity; it literally consists of predicting a variable with itself (at different timescales). Thus, when scholars consider extraversion in relation to holiday shopping (Funder & Ozer, 2019); agreeableness in relation to successful social interactions (Funder & Ozer, 2019); personality in relation to occupational attainment, social success, financial security, mortality, and even more removed predictors like climate and physical topography (Götz et al., 2022); or any other accumulation for which researchers’ favored predictors differ substantially from the outcome, then those accumulating effects are unlikely to be as substantial as in the Abelson (1985) example unless all causal forces except the favored ones happen to cancel out in the long term ( $\sigma_{E_{long}}^{2}=0$ ) and unless none of the favored ones do ( $\sigma_{M_{short}}^{2}=0$ ).

Consequently, if one is expecting one’s psychological effect to accumulate in the long term in the same consequential way as in the Abelson example, one will consistently overestimate those long-term effects.

### Consequences of unmodeled long-term variance s^2^*Elong* $\sigma_{E_{long}}^{2}$

To understand the limits of the Abelson (1985) example in greater detail, say first that the Abelson model is correct in that there is no short-term variance captured by the modeled variable that fails to generalize to the long term (meaning $\sigma_{M_{short}}^{2}=0$ ), but that the long-term outcome $Y_{1:}$ ∞ now also depends on other effects than the modeled ones (so $\sigma_{E_{long}}^{2}>0$ ).

Why is the possibility of nonmodeled stable (between-person) variance important to consider in general? Because, as noted, Abelson makes a very strict assumption about the sources of long-term variances in the dependent variable: All of it comes from the independent variable. However, we know that this is unlikely to be true in general because anyone thinking about a psychological or behavioral outcome can immediately think of multiple potential sources of variance.

In general, then, we must account for the possibility of long-term variation that we do not capture with our model. In our formulation, this is done with $\sigma_{E_{long}}^{2}$ . The unmodeled short-term variance $\sigma_{E_{short}}^{2}$ in the Abelson (1985) example is now broken up into this further $\sigma_{E_{long}}^{2}$ part that remains in the long term. The explained variance in the long term $R_{1:}^{2}$ ∞ will therefore be smaller, even if the short-term explained variance (in the single trial) remains the same. This can have stark consequences for the effect size in the long term.

First, consider the holiday-shopping example presented by Funder and Ozer (2019) and taken from Weston et al. (2019), in which the correlation is .06 between extraversion and the amount spent on holiday shopping during one season (one holiday season equals one trial).^5^ The square of this correlation is the explained variance for one trial, $R_{t}^{2}=\;.06^{2}=.0036$ . To follow the Abelson argument, we must assume that extraversion and the variance in holiday spending explained by extraversion are both stable between time points (e.g., a person 1 *SD* above the mean at one time point remains so across other time points, and this always predicts 0.06 *SD*s higher spending than average). We must also assume that no other sources of variation between people in holiday spending are stable (e.g., all other sources of variance in spending are random across people from one trial to the next). Then the explained variance in the single trial of .0036 would eventually go toward $R_{1:\infty }^{2}=1$ , or 100%, as the number of holiday seasons increases.

However, Weston et al. (2019) reported other correlations in their Table 1, including a .35 correlation between income and holiday spending. For this variable we therefore have $R_{t}^{2}=\;.35^{2}=..1125$ . Suppose income and the variance it explains are also relatively stable across holidays—say, 50% of the variance in income is stable across time points—and assume that income and extraversion are independent across participants.^6^ Then the long-term explained variance $R_{1:\infty }^{2}=$ of extraversion will be notably smaller, at $R_{1:\infty }^{2}=.0036/(.0036+.50\times .1125)\approx .060$ . That is, rather than 100%, 6% of the variance in long-term holiday spending between people would be explained by extraversion, whereas the other 94% would be explained by income. Many other possibilities also exist depending on the (in)stability of different sources of variance.

Next, using Abelson’s (1985) example as the baseline (an explained variance of .00317 for the single bat), we examine how the explained variance develops across 1,000 independent trials (the number of trials considered in the long term in Abelson, 1985). Figure 1 illustrates this under Abelson’s strict assumption (the solid line) when the remaining variance for a single trial consists solely of short-term effects that cancel out in the long term. We also include more realistic scenarios when a mere 5% and 1% of the nonmodeled variance remains in the long term. For comparison, income differences explained about 5.6% of the stable nonmodeled variance in holiday spending in the example above (i.e., variance not modeled by extraversion; 0.5×0.1125/(1−0.0036)≈0.056 ). Note that Figure 1 illustrates how the explained variance changes as the number of trials increases. It does not illustrate how, for example, the slope in the linear regression changes over time (the slope is the same at each time point) or how a variable changes over time (as in, e.g., longitudinal growth models).

**Fig. 1.**
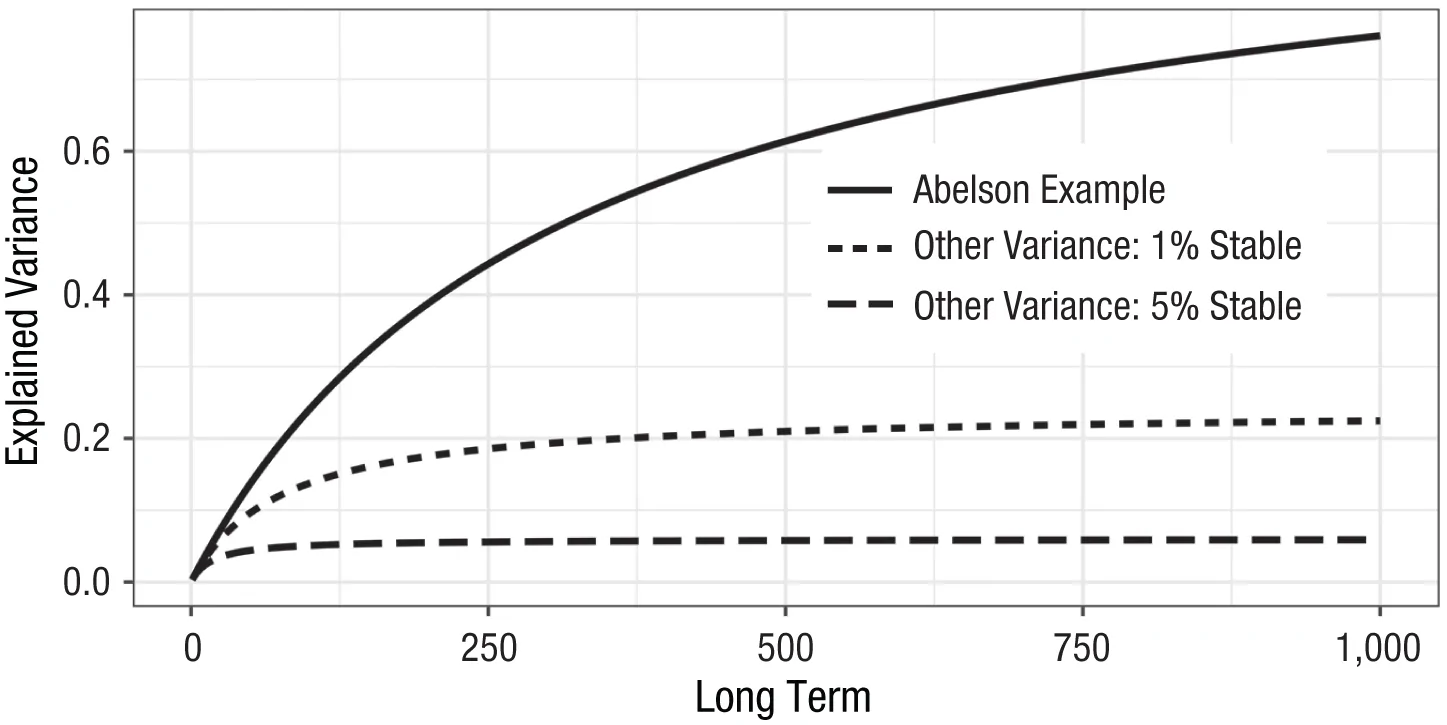
Abelson example versus other long-term effects. The solid line represents the development of the long-term explained variance in the Abelson (1985) example over 1,000 trials where there is an explained variance of .00317 for a single trial, with no unmodeled long-term variance, and where the predictor captures no short-term variance. The long-dashed line represents the same development where just 5% of the remaining variance in the single trial is explained by nonmodeled effects that hold up in the long term. The short-dashed line instead represents the development where just 1% of the remaining variance in the single trial hold up in the long term.

As can be seen in Figure 1, this inclusion leads to a dramatic drop in the accumulation of the explained variance. In the Abelson (1985) example, 1,000 trials are enough that about 75% of the long-term variance is now explained by the modeled variable. However, if only 5% of the nonmodeled variance in the single trial is due to other long-term effects (such as income on holiday spending), then the predictor has reached only about 6% explained variance. If more of the remaining variance is from other long-term effects, then this accumulation will be even less impressive. Less than 1% of the remaining variance can remain stable for the explained variance in the long term to start climbing to the same levels as in the Abelson example. Abelson levels of accumulation of small effect sizes thus require that only an extremely small amount of the unmodeled variance is stable in the long term.

The unmodeled variance need not be the result of just one other variable that remains stable across trials. As long as some nonmodeled effects remain stable, and add up to those percentages, then we would see that the accumulation of an effect size of .00317 would develop like one of the dashed lines in Figure 1, rather than the solid line representing the Abelson (1985) example.

Figure 1 illustrates how using the Abelson (1985) example to think about psychological variables will most likely severely overestimate their long-term effects. Indeed, in the very situations where the Abelson argument is attractive, where the explained variance in the short term is small, there will be more unexplained variance, and so more room for some other variable to explain additional stable variance. Even if a modest proportion of the remaining variance remains stable, the actual accumulation of researchers’ effect sizes will be nothing akin to that in the Abelson example.

See the Open Science Framework (OSF) link at the end of this article for the R code used to construct the figures.

### Consequences of modeled short-term variance s^2^_*Mshort*_ $\sigma_{M_{short}}^{2}$

There are potentially even more complications when comparing long-term to short-term psychological effects. Going back to the complete formulation of the one-time variance in Equation 1, we now add modeled short-term explained variance $\sigma_{M_{short}}^{2}$ —that is, variance from transitory (short-term) effects captured by our model.

It seems reasonable to expect such effects in many cases. Suppose the relationship between people’s self-rated extraversion and happiness is examined. Furthermore, suppose participants who happen to have had a particularly good (or bad) day tend to rate themselves as happier (or unhappier) and more (or less) interested in socializing with others (extraversion) than they would have on other days. Then the relationship is not only a result of stable long-term effects, but also of short-term state variations. Consequently, some variance captured by the modeled variable will not remain in the long term (i.e., $\sigma_{M_{short}}^{2}>0$ ).

In the examples from the previous sections, the long-term explained variances were always at least as large as the short-term explained variances. However, now that some variance is explained only in the short term, this need not be the case. The long-term explained variance in Equation 2 can therefore be attenuated compared to the short-term explained variance in Equation 1. This will happen if $\sigma_{M_{short}}^{2}/\sigma_{E_{short}}^{2}>\sigma_{M_{long}}^{2}/\sigma_{E_{long}}^{2}$ . That is, attenuation will occur if the ratio of captured to uncaptured short-term variance is larger than the ratio of captured to uncaptured long-term variance. In Figure 2, the four types of variances are varied to illustrate different types of developments. The challenge, again, is that it is not known how explained and unexplained variation will develop in the long term without actually measuring what happens in that long term.

**Fig. 2.**
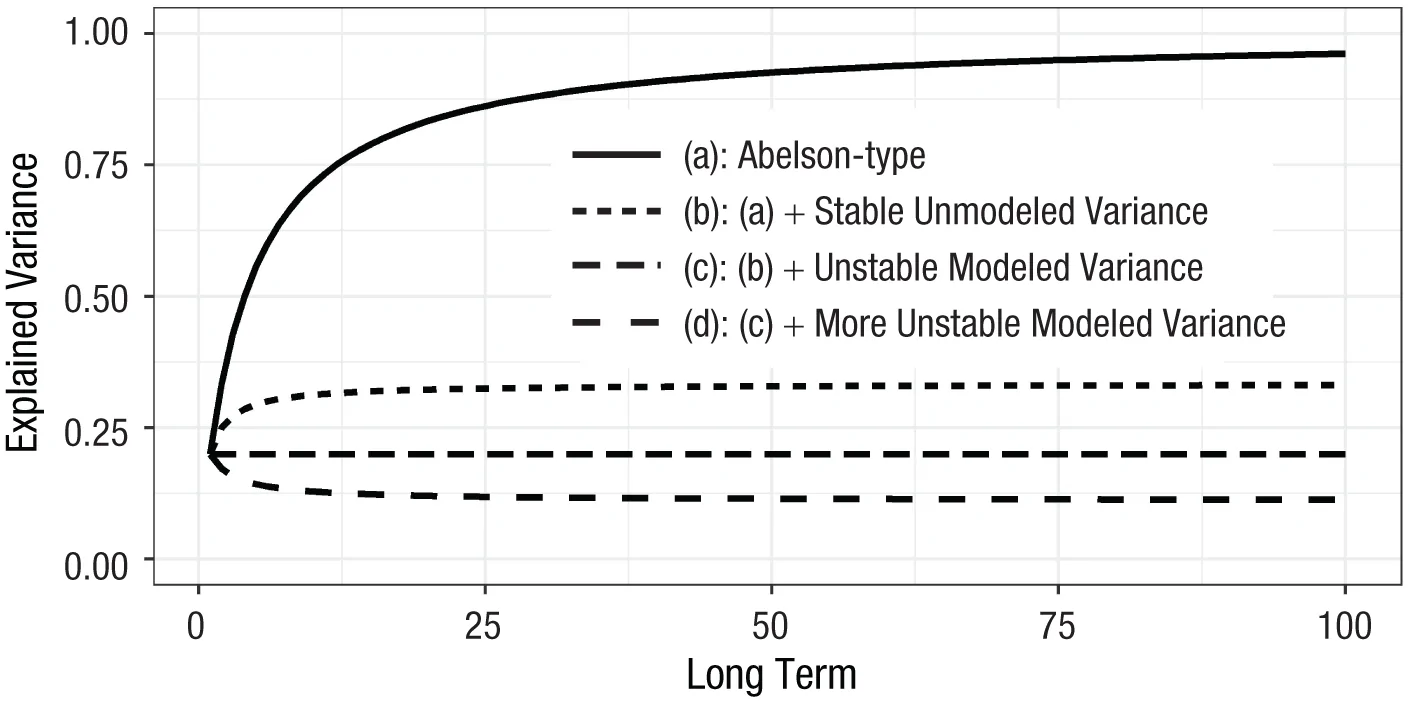
Increases, no changes, and decreases in the long term, for an initial explained variance of 20%, over 100 trials, under the same formulation as in Figure 1. In the figure, (a) through (d) are in the order of the explained variance in the long term, with (a) being the largest and (d) the smallest); $\left(\sigma_{M_{long}}^{2},\sigma_{M_{short}}^{2},\sigma_{E_{long}}^{2},\sigma_{E_{short}}^{2}\right)$ represents the long- and short-term variances (set to add up to 1 for simplicity). In (a) we show an Abelson-type example in which the modeled variance fully holds up in the long term, with no other long-term variance (.2, 0, 0, .8). In the long term, the explained variance increases to 100%. Say that extraversion explains 20% of the one-trial variance in holiday spending, with no other stable effects. In contrast, half the unmodeled variance in (b) holds up in the long term (.2,0,.4,.4,). The explained variance now only increases to 33%. Other effects from, for example, income, wealth, other expenses, frugality, and social norms may add up to this additional stable variance. In addition to (b), half the modeled variance in (c) does not survive in the long term (.1,.1,.4,.4). Now there is no change in the explained variance in the long term, compared with the short term. Say that extraversion fluctuates or that its effect varies from one holiday to the next (if some holidays target more extraverted people than others) so that not all of its effect reflects stable between-person variance. Variances in (d) are as in (c), except that only 25% of the modeled variance survives in the long term (.05,.15,.4,.4); now the explained variance decreases to 11%.

What does this mean for large effect sizes? An effect size that is large in the short term will not be much affected by the inclusion of unmodeled variance that survives in the long term ( $\sigma_{E_{long}}^{2}>0$ ). It already explains a large proportion of the variance, so if this is the only additional inclusion compared to in the Abelson example, it will continue to explain a large proportion in the long term. This gives reliably estimated large effect sizes a better starting point than small short-term effect sizes, all else being equal. However, the argument above means that even large short-term effect sizes could be small in the long term, if they are unstable enough because of a large $\sigma_{M_{short}}^{2}$ term.

## Discussion

### Model summary

Here, we considered a simple statistical model of population level (or true) long-term versus short-term explained variances, of which Abelson’s example is a special case. This revealed that if psychological researchers hold out hope that their variables will show similar accumulation in the long term as in the Abelson example (hit rate over a set number of bats predicted by average hit rate over all bats), then this will most likely overestimate the accumulation for those psychological variables. As revealed in Figure 1, this overestimation can be quite severe. If complementary short-term and long-term variances are considered, even modest additions, in the presence of weak effects in the single trial, can dramatically alter the development of the explained variance in the long term.

The main point of this statistical demonstration is also very general. That is, we demonstrate in Equations 1 and 2 how the population-explained variance in a single trial can change in the long term (i.e., the limit as T goes to infinity). Several different types of models can be captured with this decomposition. As long as the model of independent variables captures the mean trend in the dependent variable, this variance decomposition applies (see Appendix A in the Supplemental Material), although the rate of change over trials ( 1/T ) can vary. If extraversion explains an average .0036 variance in holiday spending that is stable in the long term, and income explains an independent average .50×.1125 variance that is stable in the long term, and nothing else is stable, then the (very) long-term explained variance of extraversion will be 6%, not 100% or anything close to it. This does not change even if the relationship between trials is more complex (e.g., if trials are not independent and if the effect size varies across trials; see Appendix A).

### Meaningful interpretations of effect sizes

Following the Abelson (1985) formulation, it was assumed that the effects of interest were best described with explained variances. However, this need not be the case (see Berggren, 2024, for discussion and alternative correlational effect sizes). Changing the measure will further affect how that effect size develops in the long term, compared with the short term.

It is also not certain that correlational effect sizes—which measure the proportion of variation explained in various ways—are the proper choice of quantification. For example, a result with a small effect size, often cited in this context, is a correlation of .034 between aspirin and the prevention of heart attacks (Rosenthal, 1990). However, in this case, the .034 correlation corresponds to a decrease in the probability of heart attacks from an estimated 1.71% to 0.93%. Put differently, aspirin decreases the risk of heart attacks by a full 46% (i.e., 1 – 0.0093/0.0171 ≈ 0.46). This percentage makes it much easier to understand the consequences than the correlation does, and it arguably better captures the outcome of interest for this variable: how much we can reduce heart attacks with this relatively cheap intervention (see also Primbs et al., 2023, p. 510). Note, though, that this is a very different type of argument than the one that effects can accumulate to explain more of the variation in an outcome. If the goal had been to understand as much of the variation in heart attacks as possible, then a correlational effect size would again be more appropriate, and would reveal that explaining it only through aspirin intake would remain insufficient. Here, there is relatively strong control of the modeled variable (aspirin), so whether it explains a small proportion of the variation matters little, because it can be reliably manipulated to produce a systematic effect. Even in such cases, though, one should still examine whether the effect holds for a larger group of people, does not diminish through mechanisms such as habituation or counteracting responses, and replicates across different samples (Anvari et al., 2023).

### Recommendations

We should reward research that is conducted so confounds are minimized and so the important consequences of the effects follow as directly as possible (see also Anvari et al., 2023; Funder & Ozer, 2019; Götz et al., 2022; Primbs et al., 2023). The studies of aspirin and heart attacks referenced above illustrate this: The key outcome is the proportion of heart attacks that can be prevented by prescribing aspirin, which is directly measured in these studies. When researchers examine an effect in the short term and then speculate about the effect in the long term, the risk is that those long-term consequences remain highly uncertain (as illustrated here). How exactly to align measurements with key consequences will differ depending on the research goal. If the importance lies in the long term, long-term effects should be studied. Often, this is done by studying multiple time points. When possible, it could also be done by studying outcomes that directly measure long-term consequences (e.g., longevity; see Friedman et al., 1993). Similarly, if the importance lies in behaviors, then corresponding behavioral measures should be employed. And if the importance lies in the proportion of some condition (e.g., heart attacks) that can be prevented in the population, then the effect size should measure that proportional prevention. Such close correspondence between the measurements and the research question increases the internal validity of the study and leads to more proper inferences.

This is not to say that there is no point in doing shorter-term, often simpler studies. They might demonstrate a theoretically meaningful effect with practical consequences that warrant further study. As stated in the introduction, quantifying short-time effect sizes has its own advantages, such as allowing more evidence for a nonspurious relationship. However, it should be acknowledged when the practical consequences remain uncertain, when they are only indirectly examined, or when effect sizes appear weaker than expected in the particular setting that has been observed. Aligning studies with practical consequences is required for effect sizes (large or small) to confirm or rule out the importance of effects.

Regarding the Abelson-type argument about substantial accumulation:

1. It should not be the default, as it is a highly restrictive edge case unlikely to be representative of psychological effects of real theoretical importance. It assumes:
  a. The dependent variable is not affected by other variables of long-term importance.
  b. The model does not capture effects that cancel out in the long term.

2. The onus is on researchers arguing for substantial accumulation to demonstrate that their effects truly accumulate meaningfully.
3. If a researcher argues for long-term effects in a study but does not examine it and finds support only for small short-term effects, this should be treated as a limitation of that study. Contrary to what is often done (see Anvari et al., 2023), potential long-term accumulation, to Abelson’s degree, should not be used to argue for the importance of the effect size unless it is clearly labeled as speculative and as relying on restrictive assumptions about other effects.

## Conclusions

By considering only the most flattering case, the argumentation for the accumulation of effects finally brings to mind a long-standing criticism of null hypothesis significance testing—namely, that it invites setting up weak predictions from one’s theory (e.g., that “some effect is not exactly 0”), and then taking evidence against the highly restrictive complement of those predictions (e.g., that “some effect is exactly 0”) as support specifically for that favored theory (see, e.g., Cohen, 1994; Meehl, 1990; Lykken, 1968). The arguments for accumulation seem to proceed as follows: If there is a substantial effect in the long term, then there should also be some effect in the short term (a valid modus ponens argument from affirming the antecedent, as this indeed follows logically from Equations 1 and 2).^7^ Once some evidence against a null effect in the short term is provided, this is therefore also taken as support of a meaningful long-term effect even if the short-term effect does not seem very meaningful. (This is an invalid argument from affirming the consequent, as this does not follow logically from Equations 1 and 2.^8^) This also skips the necessary step of empirically evaluating the long-term development of that effect.

There is of course nothing inherently incorrect in observing that effect sizes can be larger in the long term, or in situations in which the error variance is otherwise accounted for. The issue with the argumentation that follows the Abelson (1985) example lies in the tendency to consider *only* the possibility for larger effect sizes in the long term and not sufficiently recognize that such effect sizes may be smaller than expected or not very different from the short-term effect sizes (see also Anvari et al., 2023; Primbs et al., 2023). Arguably, the way the Abelson (1985) example is set up invites such one-sided speculation.

In sum, if the theoretical value of an effect lies in the long term, then it should be evaluated in the long term, not speculated about in the short term. Fortunately, the solution is straightforward: Study more such outcomes in the long term. Only then can the long-term importance of psychological variables be established.

## Supplemental Material

sj-docx-1-pps-10.1177_17456916261452937 – Supplemental material for Effects May Both Increase or Decrease in the Long Term: A Statistical Illustration Supplemental material, sj-docx-1-pps-10.1177_17456916261452937 for Effects May Both Increase or Decrease in the Long Term: A Statistical Illustration by Mathias Berggren and Jessica Kay Flake in Perspectives on Psychological Science

## Transparency

*Action Editor:* Arturo E. Hernandez

*Editor:* Arturo E. Hernandez

**Open Practices:** R code for the figures in this article, as well as the code for the simulations, is provided through the Open Science Foundation: https://osf.io/a6p24/. Derivations are provided in the Supplemental Material.

## Notes

1. Anvari et al. (2023) also considered further mechanisms across contexts rather than across time points, such as cascades that can cause an intervention to not only affect the participants but also their friends.

2. If the same number of time points are studied for each participant, as we always consider, then this yields the same explained variance as if we considered the sum instead of the mean.

3. This is because of the law of large numbers, which states that the mean of a large number of draws from a random variable will converge toward the true mean of that variable. Here, if bats are independent, then the probability of a hit equals the player’s true batting average, and the mean result over multiple bats will converge towards this probability.

4. Abelson (1985) also noted implications for later outcomes, such as which team has more wins over a season. However, all the mathematical formulations and arguments concern the relationship between skill level (i.e., batting averages) and one-time bats. Here we examine what happens when the long-term outcome ${\bar{Y}}_{1:}$ _∞_ depends on things other than variation captured by the model and when the model captures transient variation that cancels out in the long term. This in itself reveals that the long-term accumulation is often strongly attenuated relative to that in the Abelson (1985) example. Further, if the substantial outcome of interest is not as strongly related to the measured long-term outcome, then the ultimate effect of the modeled variable on the substantial outcome will be even more attenuated than shown here—mediation model: measured predictor (e.g., skill level) → measured outcome (e.g., batting results) → substantial but unmeasured outcome (e.g., team wins).

5. Funder and Ozer (2019) refer to this as .09. However, according to Table 1 in Weston et al. (2019), this refers to the total spending; .06 is for holiday spending.

6. In Weston et al.’s (2019) Table 1, they have a nonsignificant correlation of .04, 95% CI = [−.01, .08], so they at least appear approximately uncorrelated in their data.

7. If the explained variance in the long term is higher than zero, $R_{1:\infty }^{2}=\sigma_{M_{long}}^{2}/\left(\sigma_{M_{long}}^{2}+\sigma_{E_{long}}^{2}\right)>0$ , this means that the variance captured by the model in the long term is higher than zero, $\sigma_{M_{long}}^{2}>0$ , which implies that the explained variance in the short term is higher than zero, $R_{t}^{2}=\left(\sigma_{M_{long}}^{2}+\sigma_{M_{short}}^{2}\right)/\left(\sigma_{M_{long}}^{2}+\sigma_{M_{short}}^{2}+\sigma_{E_{long}}^{2}+\sigma_{E_{short}}^{2}\right)>0$ .

8. If the explained variance in the short term is higher than zero, $R_{t}^{2}=\left(\sigma_{M_{long}}^{2}+\sigma_{M_{short}}^{2}\right)/\left(\sigma_{M_{long}}^{2}+\sigma_{M_{short}}^{2}+\sigma_{E_{long}}^{2}+\sigma_{E_{short}}^{2}\right)>0,$ this does not imply that the explained variance in the long term is higher than zero, $R_{1:\infty }^{2}=\sigma_{M_{long}}^{2}/\left(\sigma_{M_{long}}^{2}+\sigma_{E_{long}}^{2}\right)>0$ , nor that the explained variance in the long term is much greater than the explained variance in the short term, *R*^2^_1:∞_ $\gg R_{t}^{2}$ . Perhaps the nonzero variance explained by the model is entirely due to variance in the short term: $\sigma_{M_{short}}^{2}>\sigma_{M_{long}}^{2}=0$ .
