# Supplementary material for: Effects May Both Increase or Decrease in the Long Term: A Statistical Illustration
Source: Perspect Psychol Sci. 2026 Jul 30;21(6):640–50. doi: 10.1177/17456916261452937 (PMC13714218; doi:10.1177/17456916261452937)
Supplement: Supplementary file 1 [file sj-docx-1-pps-10.1177_17456916261452937.docx]

**Supplementary Information**

*Effects may both increase or decrease in the long term: A statistical illustration*

Mathias Berggren & Jessica Kay Flake

**Appendix A**

***Statistical model***

**Model for equal effects and independent trials**

Here, we present our model of long-term and short-term explained variance in greater detail, starting with the simpler version. Under this model, the outcome/dependent variable $Y_{t,i}$ is a linear function of the model of independent variables $M_{t,i}$ (e.g., a linear model), equal to the mean of the outcome for each value of the independent variables, plus an error $\varepsilon_{t,i}$, where the subscripts indicate that this is for timepoint/trial $t$, and participant $i$:

$$\begin{aligned} Y_{t,i}=M_{t,i}+\varepsilon_{t,i}\#\left( A1 \right) \end{aligned}$$

The model in (A1) is the one researchers consider when studying a relationship between two variables at a single timepoint/trial (using e.g., a linear model $M_{t,i}=\beta_{0}+\beta_{1}X_{t,i}$). When studying effect sizes over multiple trials, the $M_{t,i}$ and $\varepsilon_{t,i}$ terms in (A1) can further be broken up into a sum of one part that is stable over trials for the same person, that is: that person’s average value over trials, $\mu_{X_{i}}$ and $\mu_{\varepsilon_{i}}$; and one part that varies randomly between trials, $Z_{t,i}$ and $\delta_{t,i}$. In particular, our assumption that trials are independent within participants means the $Z_{t,i}$ are independent from each other across trials within participants and identically distributed across trials, as are the $\delta_{t,i}$. This yields the following extension of (A1):

$$\begin{aligned} Y_{t,i}=\left( \mu_{X_{i}}+Z_{t,i} \right)+\left( \mu_{\varepsilon_{i}}+\delta_{t,i} \right)=\left( \mu_{X_{i}}+\mu_{\varepsilon_{i}} \right)+Z_{t,i}+\delta_{t,i}\#\left( A2 \right) \end{aligned}$$

The $\mu_{X_{i}}+\mu_{\varepsilon_{i}}$ part is stable across trials for the same participant, while the $Z_{t,i}+\delta_{t,i}$ part differs. Furthermore, the $\mu_{X_{i}}+Z_{t,i}$ part is captured by the modelled variable, while the $\mu_{\varepsilon_{i}}+\delta_{t,i}$ part is not.

To measure accumulating effects, we assumed the long-term outcome over $T$ timepoints, $\bar{Y}_{1:T,i}$, equals the mean of multiple short-term outcomes $Y_{t,i}$. However, formulating it as a sum, rather than as a mean, leads to the same standardized effect sizes as long as the same number of trials $T$ are observed for each unit of study (as taking the sum just multiplies all terms by $T$). Thus, the model for the long-term outcome over $T$ trials equals:

$$\bar{Y}_{1:T,i}=\frac{1}{T}\sum_{t=1}^{T} Y_{t,i}=\frac{1}{T}\sum_{t=1}^{T} \left( \left( \mu_{X_{i}}+Z_{t,i} \right)+\left( \mu_{\varepsilon_{i}}+\delta_{t,i} \right) \right)=$$

$$\begin{aligned} =\mu_{X_{i}}+\frac{1}{T}\sum_{t=1}^{T} Z_{t,i}+\mu_{\varepsilon_{i}}+\frac{1}{T}\sum_{t=1}^{T} \delta_{t,i}\#\left( A3 \right) \end{aligned}$$

The variance of the sum of two variables $A$ and $B$ equals the sum of their variances plus two times their covariance (see e.g., Gut, 2009, p.10, eq. 7.4):

$$\begin{aligned} Var\left( A+B \right)=Var\left( A \right)+2Cov\left( A,B \right)+Var\left( B \right)\#\left( A4 \right) \end{aligned}$$

Consequently, if the covariance between two variables is zero, then the variance of their sum $Var(A+B)$; equals the sum of their variances $Var\left( A \right)+Var(B)$. The covariance between two variables can be zero for several reasons. One such reason is that the expected value of one variable is the same, no matter the value of the other variable (e.g., Gut, 2009, p.10). In our model, we first have:

$$Var\left( Y_{t,i} \right)=Var\left( M_{t,i}+\varepsilon_{t,i} \right)=$$

$$\begin{aligned} =Var\left( M_{t,i} \right)+2Cov\left( M_{t,i},\varepsilon_{t,i} \right)+Var\left( \varepsilon_{t,i} \right)\#\left( A5 \right) \end{aligned}$$

The covariance between the model $M_{t,i}$, and the error $\varepsilon_{t,i}$, is zero, by usual properties of least squares regression (e.g., Johnson & Wichern, Ch. 7; note that as we assume our model captures the mean-trend, it is the least-squares optimal model). Thus, we have:

$$\begin{aligned} Var\left( Y_{t,i} \right)=Var\left( M_{t,i} \right)+Var\left( \varepsilon_{t,i} \right)\#\left( A6 \right) \end{aligned}$$

By splitting up $M_{t,i}$ and $\varepsilon_{t,i}$ into the parts from (A2) that is stable across trials, and the parts that vary, we have:

$$\begin{aligned} Var\left( M_{t,i} \right)=Var\left( \mu_{X_{i}} \right)+2Cov\left( \mu_{X_{i}},Z_{t,i} \right)+Var\left( Z_{t,i} \right)\#\left( A7 \right) \end{aligned}$$

$$\begin{aligned} Var\left( \varepsilon_{t,i} \right)=Var\left( \mu_{\varepsilon_{i}} \right)+2Cov\left( \mu_{\varepsilon_{i}},\delta_{t,i} \right)+Var\left( \delta_{t,i} \right)\#\left( A8 \right) \end{aligned}$$

$\mu_{X_{i}}$ is the expected value (mean) of $X_{t,i}$ over all trials: $E_{t}\left( X_{t,i} \right)=\mu_{X_{i}}$ (the $t$ subscript in $E_{t}$ is to clarify that this expectation is taken over trials). Therefore, the expected value of $Z_{t,i}$ is zero for each participant, so the expected value of it is also the same constant for all values of $\mu_{X_{i}}$. Thus $Cov\left( \mu_{X_{i}},Z_{t,i} \right)=0$ and (A7) simplifies to:

$$\begin{aligned} Var\left( M_{t,i} \right)=Var\left( \mu_{X_{i}} \right)+Var\left( Z_{t,i} \right)\#\left( A9 \right) \end{aligned}$$

The same applies to the terms in (A8), which therefore simplifies to:

$$\begin{aligned} Var\left( \varepsilon_{t,i} \right)=Var\left( \mu_{\varepsilon_{i}} \right)+Var\left( \delta_{t,i} \right)\#\left( A10 \right) \end{aligned}$$

Plugging these into (A6), we get:

$$\begin{aligned} Var\left( Y_{t,i} \right)=Var\left( \mu_{X_{i}} \right)+Var\left( Z_{t,i} \right)+Var\left( \mu_{\varepsilon_{i}} \right)+Var\left( \delta_{t,i} \right)\#\left( A11 \right) \end{aligned}$$

(A11) therefore provides a formula for the variance of the short-term outcome $Y_{t,i}$ in relation to the variance of four parts: the part of the model that is stable across trials for the same participant $\mu_{X_{i}}$, the part of the model that varies across trials $Z_{t,i}$, the part of the error that is stable across trials $\mu_{\varepsilon_{i}}$, and the part of the error that varies across trials $\delta_{t,i}$.

Next, we need to apply these variances to $\bar{Y}_{1:T,i}$. As (A3) expressed $\bar{Y}_{1:T,i}$ as the sum of four parts, $\mu_{X_{i}}$, $\frac{1}{T}\sum_{t=1}^{T} Z_{t,i}$, $\mu_{\varepsilon_{i}}$, and $\frac{1}{T}\sum_{t=1}^{T} \delta_{t,i}$, and as these all relate to each other in the same way as when calculating the variance of $Y_{t,i}$ (i.e., the corresponding covariances will all be zero, due to the independence between trials), we have:

$$\begin{aligned} Var\left( \bar{Y}_{1:T,i} \right)=Var\left( \mu_{X_{i}} \right)+Var\left( \frac{1}{T}\sum_{t=1}^{T} Z_{t,i} \right)+Var\left( \mu_{\varepsilon_{i}} \right)+Var\left( \frac{1}{T}\sum_{t=1}^{T} \delta_{t,i} \right)\#\left( A12 \right) \end{aligned}$$

The variances for the stable parts are the same across trials. For the parts that vary across trials, we need to consider the variance of the mean of $T$ of these terms. The variance of the mean of $T$ independent and identically distributed variables equals the variance of one of those variables divided by $T$ (e.g., Gut, 2009, p.10). We can therefore simplify (A12) to:

$$\begin{aligned} Var\left( \bar{Y}_{1:T,i} \right)=Var\left( \mu_{X_{i}} \right)+\frac{1}{T}Var\left( Z_{t,i} \right)+Var\left( \mu_{\varepsilon_{i}} \right)+\frac{1}{T}Var\left( \delta_{t,i} \right)\#\left( A13 \right) \end{aligned}$$

We can now simplify notations by removing subscripts and returning to the terms from the main text. Using the notations from the main text on the left side, and the notations above on the right, we have:

$$\begin{aligned} \bar{Y}_{1:T}=\bar{Y}_{1:T,i} \#\left( A14 \right) \end{aligned}$$

$$\begin{aligned} Y_{t}=Y_{t,i} \#\left( A15 \right) \end{aligned}$$

$$\begin{aligned} M_{long}=\mu_{X_{i}} \#\left( A16 \right) \end{aligned}$$

$$\begin{aligned} M_{short}=Z_{t,i} \#\left( A17 \right) \end{aligned}$$

$$\begin{aligned} E_{long}=\mu_{\varepsilon_{i}} \#\left( A18 \right) \end{aligned}$$

$$\begin{aligned} E_{short}=\delta_{t,i} \#\left( A19 \right) \end{aligned}$$

Which, as noted in the main text, is to be understood as the part of the model that holds in the long term $M_{long}$, the part that only holds in the short term $M_{short}$, and the part of the error that holds in the long term $E_{long}$, as well as the part of the error that only holds in the short term $E_{short}$. Plugging these into (A13) we get:

$$\begin{aligned} Var\left( \bar{Y}_{1:T} \right)=Var\left( M_{long} \right)+\frac{1}{T}Var\left( M_{short} \right)+Var\left( E_{long} \right)+\frac{1}{T}Var\left( E_{short} \right)\#\left( A20 \right) \end{aligned}$$

Which is the expression of the variance from the main text that we used to derive the explained variances, when rewriting the notation of the variances as $\sigma_{X}^{2}$ instead of $Var(X)$.

**Generalized model**

Even if the effect varies across non-independent trials, then this will not affect the general model for comparing long-term to short-term variances, as done in equation (1)-(2) and Table 1, except this has to be formulated in terms of the average variance captured by the model in the short term (when there are no varying effects across trials the average variance is just the same as the variance for each trial). Specifically, say that the variance in the outcome that the model captures is $Var\left( M_{t,i} \right)$ in trial $t$. Then write:

$$\begin{aligned} \frac{1}{T}\sum_{t=1}^{T} Var\left( M_{t,i} \right)\#\left( A21 \right) \end{aligned}$$

This is the average variance captured by the model across the T trials. Similarly, write:

$$\begin{aligned} Var\left( \frac{1}{T}\sum_{t=1}^{T} M_{t,i} \right)\#\left( A22 \right) \end{aligned}$$

This is the variance captured in the average (long-term) outcome over the same trials, by equation (A6). By usual properties of variances, we may rewrite this as:

$$\begin{aligned} Var\left( \frac{1}{T}\sum_{t=1}^{T} M_{t,i} \right)=\frac{1}{T^{2}}\left( \sum_{t=1}^{T} Var\left( M_{t,i} \right)+\sum_{t=1}^{T} \sum_{j\neq t} Cov\left( M_{t,i},M_{j,i} \right) \right)\#\left( A23 \right) \end{aligned}$$

The covariances $Cov\left( M_{t,i},M_{j,i} \right)$ in equation (A23) must be lower than or equal to $\sqrt{Var\left( M_{t,i} \right)Var\left( M_{j,i} \right)}$. This can be seen from the fact that the Pearson correlation $Cor\left( M_{t,i},M_{j,i} \right)={Cov\left( M_{t,i},M_{j,i} \right)}/{\sqrt{Var\left( M_{t,i} \right)Var\left( M_{j,i} \right)}}$ must be lower than or equal to one. Consequently, plugging these maximums into the right side of equation (A23) yields:

$$\begin{aligned} Var\left( \frac{1}{T}\sum_{t=1}^{T} M_{t,i} \right)\leq\frac{1}{T^{2}}\left( \sum_{t=1}^{T} Var\left( M_{t,i} \right)+\sum_{t=1}^{T} \sum_{j\neq t} \sqrt{Var\left( M_{t,i} \right)Var\left( M_{j,i} \right)} \right)\#\left( A24 \right) \end{aligned}$$

For any real-valued numbers *a* and *b*, we have $\left( a+b \right)\left( a-b \right)=a^{2}-b^{2}\leq a^{2}$. Write:

| $Var\left( M_{t,i} \right)Var\left( M_{j,i} \right)=\left( \frac{Var\left( M_{t,i} \right)+Var\left( M_{j,i} \right)}{2}+\frac{Var\left( M_{t,i} \right)-Var\left( M_{j,i} \right)}{2} \right)\left( \frac{Var\left( M_{t,i} \right)+Var\left( M_{j,i} \right)}{2}-\frac{Var\left( M_{t,i} \right)-Var\left( M_{j,i} \right)}{2} \right)\leq\left( \frac{Var\left( M_{t,i} \right)+Var\left( M_{j,i} \right)}{2} \right)^{2}$ | $\begin{aligned} \#\left( A25 \right) \end{aligned}$ |
| --- | --- |

Plugging this into (A24) (and using that variances are always positive to ignore absolute values), we get:

$$\begin{aligned} Var\left( \frac{1}{T}\sum_{t=1}^{T} M_{t,i} \right)\leq\frac{1}{T^{2}}\left( \sum_{t=1}^{T} Var\left( M_{t,i} \right)+\sum_{t=1}^{T} \sum_{j\neq t} \frac{Var\left( M_{t,i} \right)+Var\left( M_{j,i} \right)}{2} \right)\#\left( A26 \right) \end{aligned}$$

Each $Var\left( M_{t,i} \right)$ term is the same for the $T-1$ inner sums. Each $Var\left( M_{j,i} \right)$ term is part of $T-1$ of the outer sums. Consequently, we can multiply each by $T-1$ and remove one of the sums. Additionally, in the first sum on the right, we rewrite it as $Var\left( M_{t,i} \right)=\left( Var\left( M_{t,i} \right)+Var\left( M_{t,i} \right) \right)/2$. Thus:

| $Var\left( \frac{1}{T}\sum_{t=1}^{T} M_{t,i} \right)\leq\frac{1}{T^{2}}\left( \sum_{t=1}^{T} \frac{Var\left( M_{t,i} \right)+Var\left( M_{t,i} \right)}{2}+\left( T-1 \right)\sum_{t=1}^{T} \frac{Var\left( M_{t,i} \right)+Var\left( M_{t,i} \right)}{2} \right)=\frac{1}{T}\sum_{t=1}^{T} Var\left( M_{t,i} \right)$ | $\begin{aligned} \#\left( A27 \right) \end{aligned}$ |
| --- | --- |

Where the latter equality is found by combining the sums and rewriting $\left( Var\left( M_{t,i} \right)+Var\left( M_{t,i} \right) \right)/{2=Var\left( M_{t,i} \right)}$ again. We have now proven that the variance captured by the model for the averaged outcome over trials (on the left) is smaller than or equal to the average variance captured by the models in the single trials (on the right). Thus, we can write:

$$\begin{aligned} \lim_{T\to\infty} Var\left( \frac{1}{T}\sum_{t=1}^{T} M_{t,i} \right)=\sigma_{M_{long}}^{2}\#\left( A28 \right) \end{aligned}$$

$$\begin{aligned} \lim_{T\to\infty} \frac{1}{T}\sum_{t=1}^{T} Var\left( M_{t,i} \right)=\sigma_{M_{long}}^{2}+\sigma_{M_{short}}^{2}\#\left( A29 \right) \end{aligned}$$

The $\lim_{T\to\infty}$ term means the limit as the number of trials increase to infinity. That is, $\sigma_{M_{short}}^{2}$ is the proportion of the average modelled variance $\frac{1}{T}\sum_{t=1}^{T} Var\left( M_{t,i} \right)$ that is removed when modelling the variance in the averaged outcome in $Var\left( \frac{1}{T}\sum_{t=1}^{T} M_{t,i} \right)$. The same holds for $\sigma_{E_{long}}^{2}$ and $\sigma_{E_{short}}^{2}$. These variances sum to the variance of the outcome, as in (A12), as long as there is no correlation between the model at one timepoint and the error at another. This is fulfilled if the model takes into account the same variables at each timepoint. Say, if parental SES at time t has an effect on the outcome at that timepoint, and also has an effect at later timepoints (beyond parental SES at time t+1), then it should be included at all such timepoints. As noted in the main text, this corresponds to having the appropriate population model of the effects of the independent variables. If not, one can miss factors that lead to either a stronger or weaker relationship for one’s independent variables of interest.

This means the behavior of the explained variance in the short term versus in the limit in the long term in equations (1)-(2) does not depend on the assumptions about independent trials and equal effects in the main text. Note that we made no assumptions about how the variance behaves between trials in deriving the inequality above. Thus, for researchers to argue that their favored effects will develop in a more impressive way than suggested by the results in the main text, they have to argue that the average variance across trials, as captured by their variables, is notably larger than what is found in their specific study, while the amount of unmodelled variance is not. This would mean the researchers no longer just imagine effects in a flattering light in the unexamined long term; now they also imagine them in a flattering light in the unexamined (other potential trials) in the short term.

**Appendix B**

***Application to fair and biased coin flips***

Here, we provide a simple-to-derive unifying example to illustrate the influence of the four different sources of variance in the simple model setup.

**Baseline problem**

Participants are assigned two coins from amongst Green and Red, each with probability 1/2=0.5 (so it is e.g., possible to receive two green coins). The green coins are double-headed, yielding a probability of 1 to get Heads when flipping the coin. The red coins are regular coins with 1/2 chance for Heads and 1/2 for Tails. Researchers want to predict each person’s mean number of Heads over $T$ coin flips, scoring Heads with 1, and Tails with 0, and using the mean number of Heads for the two coins as the score. Researchers know about the properties of each colored coin and want to use the number of green coins to predict the score of each participant.

**Long-term optimal model**

First, suppose coins are never reassigned between flips and that researchers can observe both coins for each participant. This corresponds to perfect knowledge about long-term variation from the researchers’ side.

Each combination of coins: Green-Green, Green-Red, Red-Green, and Red-Red, has an equal 1/4 probability for participants. For $T=1$ flip (= trial), for participants with two red coins, there is an equal 1/4 probability to get Tails-Tails, Tails-Heads, Heads-Tail, and Heads-Heads for scores of 0, 0.5, 0.5, and 1, respectively. Thus, there is a 1/4+1/4=1/2 probability to score 0.5, and a 1/4 probability to score each of 0 and 1. With one red and one green coin, the green coin always yields Heads, while the red coin has an equal 1/2 probability for Heads and Tails, yielding a 1/2 probability each to score 0.5 and 1. For two green coins, both always yield Heads for a score of 1. The mean is the sum of each value multiplied by its probability. Over multiple flips, the mean score of people with two red coins will therefore be $0\times\frac{1}{4}+0.5\times\frac{1}{2}+1\times\frac{1}{4}=0.5$, with means $0.5\times\frac{1}{2}+1\times\frac{1}{2}=0.75$, and $1\times1=1$, for people with one red and one green coin, and people with two green coins, respectively. These probabilities and means are presented at the top of Table B1. As the number of coin flips increases, the mean score over all tosses will converge to these means for each participant (by the law of large numbers).

The overall mean score of all participants is 0.75. The variance is the sum of the squared deviations from the mean multiplied by the probabilities. The variances in scores between participants for one coin flip and for an infinite number of coin flips (i.e., the variance we converge towards as the number of flips increases) are presented below the previous numbers in Table B1. As can be noted, the variance decreases with more coin flips, as short-term variation between people cancels out over multiple flips.

Next in Table B1, we have the predictions from the best linear least squares model, with $X$ = “the number of green coins”, as the predictor. This best model is given by $0.5+0.25X$, as the mean score of everyone without a green coin ($X=0$), is 0.5, the mean score of everyone with $X=1$ green coin is 0.75, and the mean score of everyone with $X=2$ green coins is 1. The model always yields the same predictions for the same combination of coins. As the coins do not change between flips, the model yields the same predictions for each participant during each flip. Therefore, the variance captured by the model remains the same for multiple coin flips.

The explained variances can be calculated by taking the variance captured by the model and dividing it by the total variance in the scores (the outcome), for each number of coin flips. These are presented at the bottom of Table B1. The model’s explained variance is 2/6≈0.333 for one flip, meaning most variance is unexplained. However, as the number of flips increases, the remaining variance between participants is entirely explained by the model, meaning the long-term explained variance is 1. This corresponds to the Abelson (1985) example. As we develop this model further below, this will no longer be the case.

**Table B1**

*Explained variances for the first coin example*

| Outcome |  |  |  | Score |  |  |  |
| --- | --- | --- | --- | --- | --- | --- | --- |
|  | Observed1 | Observed2 | Probability to receive these coins | 0 | 0.5 | 1 | Mean |
|  | Green | Green | 1/4 | 0 | 0 | 1 | 1 |
|  |  | Red | 1/4 | 0 | 1/2 | 1/2 | 0.75 |
|  | Red | Green | 1/4 | 0 | 1/2 | 1/2 | 0.75 |
|  |  | Red | 1/4 | 1/4 | 1/2 | 1/4 | 0.5 |
|  | Variance | T=1 | $\left( 0-0.75 \right)^{2}\times\frac{1}{4}\times\frac{1}{4}+\left( 0.5-0.75 \right)^{2}\times\frac{1}{4}\times\left( \frac{1}{2}+\frac{1}{2}+\frac{1}{2} \right)+\left( 1-0.75 \right)^{2}\times\frac{1}{4}\times\left( \frac{1}{4}+\frac{1}{2}+\frac{1}{2}+1 \right)=6/64$ | | | | |
|  |  | T=∞ | $\left( 0.5-0.75 \right)^{2}\times\frac{1}{4}+\left( 0.75-0.75 \right)^{2}\times\left( \frac{1}{4}+\frac{1}{4} \right)+\left( 1-0.75 \right)^{2}\times\frac{1}{4}=2/64$ | | | | |
| Model |  |  |  | Prediction |  |  |  |
|  | Observed1 | Observed2 | Probability to receive these coins | 0.5 | 0.75 | 1 | Mean |
|  | Green | Green | 1/4 | 0 | 0 | 1 | 1 |
|  |  | Red | 1/4 | 0 | 1 | 0 | 0.75 |
|  | Red | Green | 1/4 | 0 | 1 | 0 | 0.75 |
|  |  | Red | 1/4 | 1 | 0 | 0 | 0.5 |
|  | Variance | T=1 | $\left( 0.5-0.75 \right)^{2}\times\frac{1}{4}+\left( 0.75-0.75 \right)^{2}\times\frac{1}{4}\times\left( 1+1 \right)+\left( 1-0.75 \right)^{2}\times\frac{1}{4}=2/64$ | | | | |
|  |  | T=∞ | $\left( 0.5-0.75 \right)^{2}\times\frac{1}{4}+\left( 0.75-0.75 \right)^{2}\times\frac{1}{4}\times\left( 1+1 \right)+\left( 1-0.75 \right)^{2}\times\frac{1}{4}=2/64$ | | | | |
| $R_{1:1}^{2}=2/6\approx0.333$  $R_{1:\infty}^{2}=2/2=1$  Variance decomposition: $\sigma_{M_{long}}^{2}=2/64$, $\sigma_{M_{short}}^{2}=0$, $\sigma_{E_{long}}^{2}=0$, $\sigma_{E_{short}}^{2}=4/64$ | | | | | | | |
| *Note*. “Mean” is for the mean result of infinite trials for someone who received that combination of coins in the first trial. | | | | | | | |

**Unmodelled long-term variation**

Now, suppose researchers could only observe the color of one coin per participant. This corresponds to the researchers having some knowledge about factors that contribute to long-term variation between participants, although this knowledge is imperfect as there is additional variation that is unaccounted for in their model.

Everything else remains the same in relation to the example in the previous section. The probabilities, means, variances, and explained variances are presented in Table B2 below. Changes from Table B1 are highlighted in grey. As only one coin can be observed, the best linear least squares model is $0.625+0.25X$, as the mean score of everyone whose first coin is red is $(0.5+0.75)/2=0.625$, and as the mean score of everyone whose first coin is green is $(1+0.75)/2=0.875$.

Here, the explained variance is half of what it was before both for $T=1$ coinflip and as the number of flips increases. This is natural, as we were previously able to model the effect of two coins and can now only model the effect of one. Consequently, with more flips, the explained variance converges to a maximum of 0.5, rather than 1 as before. This is because there is now long-term variance between participants that the model does not capture.

**Table B2**

*Explained variances for the second coin example*

| Outcome |  |  |  | Score |  |  |  |
| --- | --- | --- | --- | --- | --- | --- | --- |
|  | Observed | Unobserved | Probability to receive these coins | 0 | 0.5 | 1 | Mean |
|  | Green | Green | 1/4 | 0 | 0 | 1 | 1 |
|  |  | Red | 1/4 | 0 | 1/2 | 1/2 | 0.75 |
|  | Red | Green | 1/4 | 0 | 1/2 | 1/2 | 0.75 |
|  |  | Red | 1/4 | 1/4 | 1/2 | 1/4 | 0.5 |
|  | Variance | T=1 | $\left( 0-0.75 \right)^{2}\times\frac{1}{4}\times\frac{1}{4}+\left( 0.5-0.75 \right)^{2}\times\frac{1}{4}\times\left( \frac{1}{2}+\frac{1}{2}+\frac{1}{2} \right)+\left( 1-0.75 \right)^{2}\times\frac{1}{4}\times\left( \frac{1}{4}+\frac{1}{2}+\frac{1}{2}+1 \right)=6/64$ | | | | |
|  |  | T=∞ | $\left( 0.5-0.75 \right)^{2}\times\frac{1}{4}+\left( 0.75-0.75 \right)^{2}\times\left( \frac{1}{4}+\frac{1}{4} \right)+\left( 1-0.75 \right)^{2}\times\frac{1}{4}=2/64$ | | | | |
| Model |  |  |  | Prediction |  |  |  |
|  | Observed | Unobserved | Probability to receive these coins | 0.625 | 0.875 |  | Mean |
|  | Green | Green | 1/4 | 0 | 1 |  | 1 |
|  |  | Red | 1/4 | 0 | 1 |  | 0.75 |
|  | Red | Green | 1/4 | 1 | 0 |  | 0.75 |
|  |  | Red | 1/4 | 1 | 0 |  | 0.5 |
|  | Variance | T=1 | $\left( 0.625-0.75 \right)^{2}\times\frac{1}{4}\times\left( 1+1 \right)+\left( 0.875-0.75 \right)^{2}\times\frac{1}{4}\times\left( 1+1 \right)=1/64$ | | | | |
|  |  | T=∞ | $\left( \frac{0.5+0.75}{2}-0.75 \right)^{2}\times\frac{1}{4}\times\left( 1+1 \right)+\left( \frac{1+0.75}{2}-0.75 \right)^{2}\times\frac{1}{4}\times\left( 1+1 \right)=1/64$ | | | | |
| $R_{1:1}^{2}=1/6\approx0.167$  $R_{1:\infty}^{2}=1/2=0.5$  Variance decomposition: $\sigma_{M_{long}}^{2}=1/64$, $\sigma_{M_{short}}^{2}=0$, $\sigma_{E_{long}}^{2}=1/64$, $\sigma_{E_{short}}^{2}=4/64$ | | | | | | | |
| *Note*. “Mean” is for the mean result of infinite trials for someone who received that combination of coins in the first trial. Changes from Table B1 are highlighted in grey. | | | | | | | |

**Modelled short-term variation**

Now, in addition to the complication in the previous section, assume that the coin that researchers can observe is changed between coin flips. This means the researchers’ idea about what contributes to long-term variation across participants is incorrect. Everything else remains the same as in the previous example.

The probabilities, means, variances, and explained variances are presented in Table B3 below. Changes from Table B2 are highlighted in grey. As in the previous section, the best linear least-squares model for one trial is $0.625+0.25X$, as the means of people whose first coin is red and people whose first coin is green remain the same. However, as the observed coin is changed between trials, the variance that the model can capture will vary between participants from one trial to the next. Thus, the effect of this variable will cancel out in the long term as everyone gathers up a similar proportion of observable red and green coins and gets the same mean result from them.

Consequently, although the explained variance in the single trial is the same as in the previous section, as the number of trials increases, the explained variance of our modelled variable now decreases and goes to zero.

**Table B3**

*Explained variances for the third coin example*

| Outcome |  |  |  | Score |  |  |  |
| --- | --- | --- | --- | --- | --- | --- | --- |
|  | Observed | Unobserved | Probability to receive these coins | 0 | 0.5 | 1 | Mean |
|  | Green | Green | 1/4 | 0 | 0 | 1 | 0.875 |
|  |  | Red | 1/4 | 0 | 1/2 | 1/2 | 0.625 |
|  | Red | Green | 1/4 | 0 | 1/2 | 1/2 | 0.875 |
|  |  | Red | 1/4 | 1/4 | 1/2 | 1/4 | 0.625 |
|  | Variance | T=1 | $\left( 0-0.75 \right)^{2}\times\frac{1}{4}\times\frac{1}{4}+\left( 0.5-0.75 \right)^{2}\times\frac{1}{4}\times\left( \frac{1}{2}+\frac{1}{2}+\frac{1}{2} \right)+\left( 1-0.75 \right)^{2}\times\frac{1}{4}\times\left( \frac{1}{4}+\frac{1}{2}+\frac{1}{2}+1 \right)=6/64$ | | | | |
|  |  | T=∞ | $\left( 0.625-0.75 \right)^{2}\times\frac{1}{4}\times\left( \frac{1}{4}+\frac{1}{2}+\frac{1}{2}+\frac{1}{4}+\frac{1}{2} \right)+\left( 0.875-0.75 \right)^{2}\times\frac{1}{4}\times\left( \frac{1}{2}+\frac{1}{2}+1 \right)=1/64$ | | | | |
| Model |  |  |  | Prediction |  |  |  |
|  | Observed | Unobserved | Probability to receive these coins | 0.625 | 0.875 |  | Mean |
|  | Green | Green | 1/4 | 0 | 1 |  | 0.875 |
|  |  | Red | 1/4 | 0 | 1 |  | 0.625 |
|  | Red | Green | 1/4 | 1 | 0 |  | 0.875 |
|  |  | Red | 1/4 | 1 | 0 |  | 0.625 |
|  | Variance | T=1 | $\left( 0.625-0.75 \right)^{2}\times\frac{1}{4}\times\left( 1+1 \right)+\left( 0.875-0.75 \right)^{2}\times\frac{1}{4}\times\left( 1+1 \right)=1/64$ | | | | |
|  |  | T=∞ | $\left( \frac{0.875+0.625}{2}-0.75 \right)^{2}\times\frac{1}{4}\times\left( 1+1 \right)+\left( \frac{0.875+0.625}{2}-0.75 \right)^{2}\times\frac{1}{4}\times\left( 1+1 \right)=0$ | | | | |
| $R_{1:1}^{2}=1/6\approx0.167$  $R_{1:\infty}^{2}=0/1=0$  Variance decomposition: $\sigma_{M_{long}}^{2}=0$, $\sigma_{M_{short}}^{2}=1/64$, $\sigma_{E_{long}}^{2}=1/64$, $\sigma_{E_{short}}^{2}=4/64$ | | | | | | | |
| *Note*. “Mean” is for the mean result of infinite trials for someone who received that combination of coins in the first trial. Changes from Table B2 are highlighted in grey. | | | | | | | |

**References**

Gut, A. (2009). *An intermediate course in probability* (2. ed.). New York: Springer Science+Business Media.

Johnson, R., & Wichern, D. (2014). *Applied multivariate statistical analysis* (6^th^ international ed.). Harlow: Pearson Education Ltd.
